# Cognitive Changes in Pre‐ataxic Spinocerebellar Ataxias: A Scoping Review

**DOI:** 10.1002/mdc3.70215

**Published:** 2025-07-14

**Authors:** Renata Barreto Tenorio, Andressa Aline Vieira, Hélio Afonso Ghizoni Teive, Carlos Henrique Ferreira Camargo

**Affiliations:** ^1^ Neurological Diseases Group, Postgraduate Program in Internal Medicine, Internal Medicine Department Hospital de Clínicas, Federal University of Paraná Curitiba Brazil; ^2^ Neurogenetic Service, Instituto de Neurologia de Curitiba Curitiba Brazil; ^3^ Movement Disorders Sector, Neurology Service, Internal Medicine Department Hospital de Clínicas, Federal University of Paraná Curitiba Brazil

**Keywords:** spinocerebellar ataxias, cognition disorders, neuropsychological tests, biomarkers, Disease Progression

## Abstract

**Background:**

Although traditionally recognized for motor impairment, evidence suggests that cognitive deficits may emerge before ataxia onset in autosomal dominant spinocerebellar ataxias (SCA), particularly in nucleotide repeat expansion SCAs (NRE‐SCAs). However, the nature and extent of these early cognitive changes and cognition disorders remain unclear.

**Objective:**

This scoping review maps existing evidence on cognitive alterations in pre‐ataxic NRE‐SCAs, focusing on affected cognitive domains, assessment tools, and early biomarkers.

**Methods:**

A comprehensive search in PubMed, EMBASE, and CINAHL was performed in accordance with Joanna Briggs Institute guidelines. Studies were included if they assessed cognitive function in genetically confirmed pre‐ataxic NRE‐SCA individuals using neuropsychological tests, imaging, or biomarkers. Data extraction comprised study design, cognitive domains assessed, and key findings.

**Results:**

Thirteen studies met inclusion criteria, examining pre‐ataxic individuals with SCA1, SCA2, SCA3, and SCA36. Executive dysfunction, particularly in cognitive flexibility, inhibitory control, and working memory, was the most frequent finding, assessed using the Trail Making Test, Stroop Test, and phonemic fluency tasks. Processing speed deficits were also commonly reported. Pre‐ataxic SCA2 exhibited the most consistent impairments, whereas findings in pre‐ataxic SCA1 and pre‐ataxic SCA3 were variable. Neuroimaging studies revealed early cerebellar microstructural changes linked to cognitive dysfunction.

**Conclusion:**

Cognitive impairments may precede motor symptoms in NRE‐SCAs, particularly SCA2. However, methodological heterogeneity and small sample sizes limit definitive conclusions. Standardized assessments and longitudinal studies are needed to clarify cognitive decline trajectories and their potential as early biomarkers.

Autosomal dominant spinocerebellar ataxias, known as spinocerebellar ataxias (SCA), represent a diverse group of neurodegenerative disorders primarily affecting the cerebellum and its associated pathways. The prevalence of SCAs is estimated at ~4 per 100,000 individuals. These disorders are genetically and clinically heterogeneous, with more than 50 subtypes caused by mutations in more than 40 distinct genes. SCA3, also known as Machado–Joseph disease, is the most common form worldwide, although regional variations exist.^1^, ^2^, ^3^ The category of polyglutamine (polyQ) expansion diseases, which includes SCAs such as SCA1, 2, 3, 6, 7, 8, 12, 17, and 51, is characterized by CAG nucleotide repeat expansions (NRE) in their coding regions. These expansions lead to the production of toxic protein tracts. Other types of NREs can also cause SCAs (NRE‐SCAs—Table S1), such as SCA36, associated with an intronic GGCCTG hexanucleotide repeat expansion in the *NOP56* gene.^3^, ^4^

Traditionally, SCAs are characterized by cerebellar dysfunction, manifesting as ataxia, dysarthria, unsteady gait, and progressive loss of motor coordination. Additionally, these disorders often present with nonmotor symptoms like cognitive impairment and retinal degeneration, which vary across the subtypes.^5^ Recent studies have highlighted the cerebellum's crucial role in cognitive processes, with SCAs exhibiting a spectrum of cognitive dysfunctions, ranging from mild dysexecutive syndromes to severe dementia, depending on the specific subtype and extent of neuropathological involvement.^5^, ^6^

In the pre‐ataxic stage, individuals carrying pathogenic mutations associated with SCAs might appear clinically unaffected in motor function although already experiencing subtle cognitive changes. Pre‐ataxic individuals are defined as genetically confirmed carriers of pathogenic SCA mutations who do not yet exhibit overt cerebellar ataxia but may present with very subtle or subclinical motor signs. There is growing evidence that cognitive decline in SCAs may begin earlier than traditionally recognized, with several studies reporting cognitive alterations even in pre‐ataxic individuals. However, research in this area remains limited, and findings are often inconsistent.^7^ These early cognitive impairments' precise scope and nature are still poorly understood. Detecting cognitive changes at this stage could yield valuable insights into the underlying neurodegenerative mechanisms and create opportunities for early therapeutic interventions.

Despite significant interest, the literature on cognitive changes in the pre‐ataxic stages of SCAs is fragmented, and to date, no scoping reviews have specifically synthesized this evidence. This scoping review aims to synthesize the existing research on cognition disorders and cognitive changes in individuals with pre‐ataxic NRE‐SCAs, identifying the types of cognitive impairments, the assessment tools employed, and potential early biomarkers of cognitive decline.

## Patients and Methods

A preliminary search was conducted on JBISRIR, the Cochrane Database of Systematic Reviews, and PubMed to identify existing scoping reviews on the topic. No previous scoping reviews specifically addressing this topic were found. The protocol for this review was registered on the Open Science Framework and is available at https://osf.io/mdtqs/?view_only=bbf8f66ec00e454786ca6635376cf1d6.

The methodology for this scoping review adheres to the Joanna Briggs Institute (JBI) framework for scoping reviews and incorporates the Preferred Reporting Items for Systematic Reviews and Meta‐Analyses extension for Scoping Reviews checklist.^8^, ^9^

## Inclusion Criteria

### Participants

Studies involved individuals with genetically confirmed NRE‐SCAs who are in the pre‐ataxic stage—those who carry the genetic mutation but do not exhibit clinical motor symptoms of ataxia.

### Concept of Interest

The focus of this review is on cognitive changes in pre‐ataxic NRE‐SCAs. This consists of studies that (1) identify specific cognitive impairments (eg, executive dysfunction, memory loss) in pre‐ataxic individuals; (2) utilize neuropsychological testing, cognitive biomarkers, or imaging studies to assess cognitive function in pre‐ataxic individuals; and (3) examine cognitive changes as they relate to disease progression, including longitudinal progression and early neurodegeneration in this population.

### Context

The scope of this review includes studies conducted in diverse settings, including clinical trials, cohort studies, observational studies, longitudinal assessments, and case reports. Studies involving individuals of any age, gender, or geographical location were considered, provided they were identified as being in the pre‐ataxic stage of NRE‐SCAs.

### Types of Sources

This scoping review incorporates original research articles, including observational, cohort, cross‐sectional, and case–control studies, that contribute to understanding cognitive changes in the pre‐ataxic stages of NRE‐SCAs. Case reports were excluded.

### Exclusion Criteria

Studies were excluded if they (1) focused exclusively on motor symptoms without addressing cognitive changes; (2) included symptomatic individuals (ataxic stage), (3) were review articles, case reports, editorials, letters, and conference abstracts without sufficient data; and (4) were not published in English.

### Search Strategy

This review focused on published primary studies. The time frame of publication was not restricted; however, only studies published in English were considered for inclusion.

A comprehensive search strategy was used to identify relevant literature. A 3‐step search strategy was utilized.

### Initial Search

A preliminary search was conducted in key databases, including PubMed, EMBASE, and CINAHL, using terms like “SCA” and “cognitive changes.” This step involved identifying relevant index terms, Medical Subject Headings, and keywords appearing in the titles and abstracts of the retrieved papers.

### Full Search

Building on the initial findings, a comprehensive second search was carried out across the same databases. This expanded search utilized all previously identified keywords and indexed terms, with the addition of terms such as “executive function,” “early neurodegeneration,” and “cognitive impairment.”

### Manual Search

To ensure thorough coverage, the reference lists of all identified studies were manually searched to uncover any additional studies that might not have been included in the database searches.

### Study Selection

The initial screening of articles was conducted using Rayyan,^10^ where titles and abstracts were evaluated against the predefined inclusion and exclusion criteria. Full‐text versions of studies that passed this preliminary screening were then obtained. These full texts were meticulously reviewed, and any studies that did not meet the criteria upon full‐text evaluation were excluded from the final selection. The literature was screened by 2 independent reviewers (R.B.T. and A.A.V.). Disagreements were resolved through discussion, and if consensus could not be reached, an additional reviewer (C.H.F.C.) was consulted. The same process was used to screen gray literature.

When a correction paper was identified for an included original study, the correction was not counted as a separate article in the final number of studies included. However, the content of the correction was reviewed and incorporated into the data extraction process to ensure the accuracy and completeness of the data. Any changes to the original data resulting from these corrections were documented, and the corrected data were used in the analysis where applicable.

### Data Charting

A structured data extraction framework was developed following JBI recommendations and adapted to meet this study's specific objectives. In addition to extracting data from the primary texts of included studies, supplementary materials were reviewed when available. Furthermore, corresponding authors of selected studies were contacted via email to request additional data or clarifications, and 2 authors provided unpublished data relevant to this review.

Data were systematically extracted across 4 key domains: (1) study identification details (article title, authors' country of origin, year of publication); (2) methodological characteristics (study design, research questions, pre‐ataxic sample characteristics, cognitive assessment methods); (3) primary findings (cognitive domains impacted, specific cognitive changes, early neurodegenerative markers); and (4) study conclusions. The presentation of data will involve a narrative summary complemented by tables that systematically organize study characteristics, cognitive domains assessed, assessment tools used, and key findings, enabling effective cross‐study comparison.

This data extraction approach is iterative, with the extraction tool reviewed and refined throughout the study to ensure comprehensive capture of relevant data, adhering to JBI guidelines.^11^

### Data Presentation

The results will be presented in a narrative summary, supported by tables and charts where appropriate. They will summarize cognitive changes, cognitive domains affected, and associated features of cognitive impairments in pre‐ataxic SCA patients, as well as highlight gaps in the literature.

## Results

### Study Selection

**FIG. 1:**
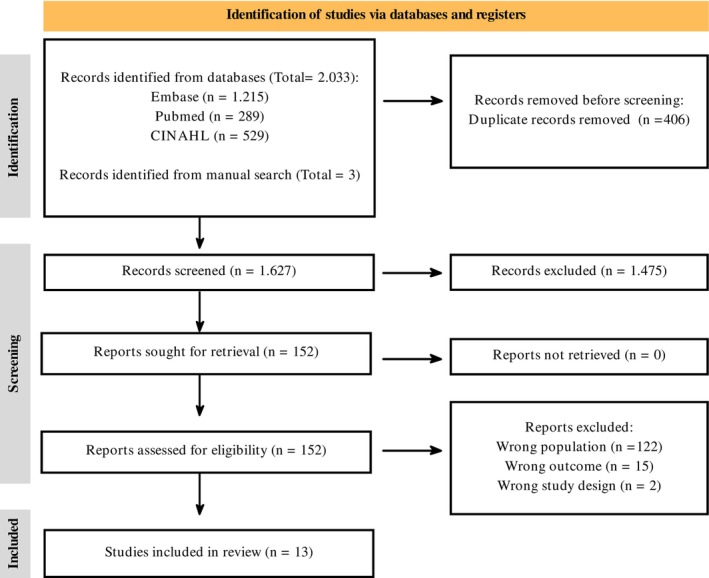
Flowchart of the studies included in the scope review.

Study selection is shown in Figure FIG. 1.^12^ Initially, 1627 nonduplicated studies were identified through database searches. An additional 3 studies were found through manual searches of reference lists. After titles and abstracts were screened, 152 studies were deemed relevant for full‐text review. Of these, only 13 studies met the stringent inclusion criteria and proceeded to the data extraction phase. These selected studies are summarized in Table 1 and detailed further in Table S2.

**TABLE 1:** Summary of characteristics and neurocognitive findings for included studies

First author (year)	Pre‐ataxic SCAs	Main cognitive findings for pre‐ataxic SCAs
Nigri (2020)^13^	13 pre‐ataxic SCA2	Lower processing speed and attention versus controls.* No decline over 1 year.
Tezenas du Montcel (2023)^14^	14 pre‐ataxic SCA1 36 pre‐ataxic SCA3	Better cerebellar cognition than symptomatics.*
Selvadurai (2024)^15^	11 pre‐ataxic SCA1 15 pre‐ataxic SCA3	Better cerebellar cognition than symptomatics.*
Velázquez‐Pérez (2014)^16^	37 pre‐ataxic SCA2	Worse executive function, fluency, memory, flexibility. Shorter TTO linked to poorer executive function, flexibility, memory; older age linked to poorer flexibility, memory.*
Bolzan (2024)^17^	35 pre‐ataxic SCA3	Subtle cognitive impairments. Shorter time to ataxia onset was associated with greater impairments than longer time to onset.
Rodríguez‐Labrada (2019)^18^	20 pre‐ataxic SCA2	Executive dysfunction.*
Wu (2017)^19^	23 pre‐ataxic SCA3	No significant differences in cognitive performance.
Ye (2024)^20^	30 pre‐ataxic SCA3	Better visual memory but worse visuospatial performance, processing speed, and memory retrieval.^a^
Elyoseph, 2024^21^	5 pre‐ataxic SCA3	Normal mentalizing.
Martínez‐Regueiro (2020)^22^	11 pre‐ataxic SCA36	Impaired phonological fluency; deficits in executive function and processing speed*; global cognition intact.
Hengel (2023)^23^	43 pre‐ataxic SCA3	Global cognition preserved.
Klinke (2010)^24^	2 pre‐ataxic SCA1	Global and emotional cognition preserved.
Li (2024)^25^	24 pre‐ataxic SCA3	Rare cognitive impairment (N = 2).^a^

*Note*: *P < 0.05; the raw data were sent by the corresponding authors upon request.

Abbreviations: SCA, spinocerebellar ataxia; TTO, time to onset of ataxia.

^a^ Indicates unpublished data.

### Characteristics of Included Studies

The 13 studies included in this review span multiple geographical regions, namely Western Europe, Brazil, China, Israel, Cuba, and the United States, with publication dates ranging from 2010 to 2024. Most studies adopted a cross‐sectional design to assess cognitive, motor, and clinical measures at a single point in time. However, certain studies implemented longitudinal designs to observe disease progression and identify early cognitive markers in SCA1 and SCA3.^13^, ^14^, ^15^ These longitudinal studies provided insights into the progression of symptoms over time. Observational studies were also prominent, gathering extensive clinical, genetic, and neuropsychological data that enriched the understanding of SCAs' natural history.^14^, ^16^, ^17^ Some employed case–control designs to contrast SCA patients with healthy controls (HC), highlighting significant cognitive and clinical differences.^18^, ^19^, ^20^

The studies primarily aimed to characterize cognitive and affective deficits across various stages of the disease.^13^, ^15^, ^16^, ^17^, ^21^, ^22^ Additionally, some research explored potential biomarkers, such as the Cerebellar Cognitive Affective Syndrome Scale (CCAS‐S) and neurofilament light chain, to assess cognitive impairments and monitor disease progression.^14^, ^15^, ^17^ Other studies examined the relationship between motor and nonmotor symptoms, investigating how the severity of ataxia correlates with cognitive dysfunctions, sleep disturbances, and depressive symptoms.^17^, ^20^, ^23^, ^24^, ^25^ Neuroimaging studies focused on the connections between brain structures and cognitive functions, particularly analyzing how cerebellar atrophy impacts cognitive performance.^13^, ^19^, ^20^, ^24^

### Assessment Tools and Participant Characteristics

Neuropsychological assessments served as the principal tools for cognitive evaluation in the reviewed studies. Executive function was primarily assessed using category switching, the Trail Making Test (TMT), and the Frontal Assessment Battery.^16^, ^17^, ^22^ Semantic and phonemic fluency tasks were employed to evaluate language abilities.^17^, ^18^, ^22^ Memory functions were assessed using tools like the California Verbal Learning Test‐Second Edition (CVLT‐II) and the Rey Auditory‐Verbal Learning Test.^16^, ^18^, ^20^, ^24^ Processing speed was measured using the Stroop Test.^16^, ^17^, ^18^, ^22^

Social cognition and affective symptoms were conducted using instruments like the Beck Depression Inventory, Hamilton Depression Rating Scale, Hamilton Anxiety Rating Scale, and the CCAS‐S. The latter evaluates a broad spectrum of cognitive domains, including executive function, attention, visuospatial processing, language, and affective symptoms.^15^, ^17^, ^20^, ^21^, ^22^ Additionally, Ye et al.^20^ applied the Minimal Assessment of Cognitive Function in Multiple Sclerosis (MACFIMS), a previously validated tool for SCA3, to comprehensively evaluate memory, processing speed, executive function, and visuospatial abilities in pre‐ataxic individuals.

Clinical scales like the Scale for the Assessment and Rating of Ataxia and the Inventory of Non‐Ataxia Symptoms were widely used to assess ataxia severity and nonmotor symptoms.^13^, ^14^, ^15^, ^16^, ^17^, ^19^, ^20^, ^22^, ^23^, ^24^, ^25^ Some studies employed neuroimaging techniques, including voxel‐based morphometry and automated cerebellar segmentation, to analyze cerebellar structural changes.^13^, ^19^, ^20^ Polysomnography was used in selected studies to assess sleep disturbances, and statistical analyses examined associations between cognitive impairment, disease severity, and other clinical variables.^16^, ^18^

Participants were categorized as symptomatic (ataxic) patients, pre‐ataxic individuals, and HCs, facilitating comparisons across different stages of the diseases. Sample sizes varied from 2 to 50 participants. Some studies included individuals far from onset (16–18 years, eg, Nigri et al.^13^; Rodríguez‐Labrada et al.^18^), whereas others focused on individuals closer to symptom onset (~6 years, eg, Tezenas du Montcel et al.^14^). Bolzan et al.^17^ stratified presymptomatic individuals into near‐onset (<4 years) and far‐from‐onset (>4 years) groups.

### Main Cognitive Findings

Cognitive impairments across different NRE‐SCA subtypes varied in severity and affected domains.

In pre‐ataxic SCA1, no significant cognitive deficits were detected compared to controls. Selvadurai et al.^15^ reported no differences in CCAS‐S scores, and Klinke et al.^24^ found normal cognitive, emotional, and motor functions in 2 asymptomatic carriers. However, Tezenas du Montcel et al.^14^ observed extracerebellar signs in pre‐ataxic patients, suggesting that neurological alterations may precede overt cognitive deficits.

In pre‐ataxic SCA2, cognitive impairments were more consistent and pronounced. Executive dysfunction, reduced processing speed, and verbal fluency deficits emerged as hallmark features.^16^, ^18^ Executive impairments particularly involved cognitive flexibility, inhibitory control, and set‐shifting, as evidenced by prolonged reaction times and increased errors in the Stroop Interference Task, impaired rule‐shifting in the Intradimensional/Extradimensional Set Shift (IED) Test (CANTAB), and reduced word generation in phonemic verbal fluency tasks.^16^, ^18^ Lower INECO Frontal Screening scores indicated early dysfunction in fronto‐cerebellar and fronto‐striatal circuits.^16^ Cognitive slowing was another key feature, with the Symbol Digit Modalities Test (SDMT) revealing reduced processing speed.^13^ Visual memory deficits were also observed, as demonstrated by increased errors and reduced correct responses in the Delayed Matching to Sample (DMS) Task, although verbal memory findings were mixed. Importantly, these impairments correlated with the estimated time to ataxia onset, suggesting a potential predictive role for cognitive testing. Sleep spindle density reductions were additionally linked to poorer verbal memory performance, indicating early sleep‐related neurophysiological changes.^16^, ^18^

In pre‐ataxic SCA3, findings were more heterogeneous. Whereas Selvadurai et al.^15^ reported no significant cognitive deficits, Bolzan et al.^17^ found intermediate cognitive performance between controls and symptomatic patients, suggesting a progressive decline. Ye et al.^20^ observed preserved verbal memory but identified superior visual memory performance (Brief Visuospatial Memory Test ‐ Revised (BVMT‐R), *P* < 0.0001) and deficits in visuospatial processing (Judgment of Line Orientation Test (JLO), *P* < 0.0001) and processing speed (Paced Auditory Serial Addition Test ‐ 3 seconds (PASAT‐3s): *P* = 0.004, Paced Auditory Serial Addition Test ‐ 2 seconds (PASAT‐2s): *P* = 0.017). Memory retrieval alterations (Clustered Recall (CR) insertions, *P* = 0.001) were also observed, despite no significant differences found in CVLT‐II, COWAT (Controlled Oral Word Association Test), MMSE (Mini‐Mental State Examination), or MoCA (Montreal Cognitive Assessment).^20^ Additionally, Wu et al.^19^ detected microstructural white matter changes in pre‐ataxic SCA3 carriers, suggesting early connectivity disruptions before clinical cognitive impairments become evident.

In pre‐ataxic SCA36, mild cognitive deficits were reported, particularly in executive function and phonemic fluency.^22^ Depressive symptoms were occasionally observed but were more pronounced among symptomatic individuals.

Overall, cognitive profiles in pre‐ataxic SCAs were heterogeneous. Pre‐ataxic SCA2 individuals consistently exhibited significant executive dysfunction, slowed processing speed, and verbal fluency deficits, often worsening as ataxia onset approached.^13^, ^16^, ^18^ In contrast, pre‐ataxic SCA1 individuals typically preserved cognitive function, suggesting a slower or distinct neurodegenerative course.^14^, ^15^, ^24^ Pre‐ataxic SCA3 and SCA36 carriers exhibited more variable findings, with some evidence of visuospatial deficits and subtle executive impairments but without the consistent profiles observed in SCA2.^14^, ^15^, ^17^, ^19^, ^20^, ^21^, ^22^, ^23^, ^25^ These differences underline the subtype‐specific nature of cognitive involvement in NRE‐SCAs, even before motor symptoms manifest.

### Longitudinal Changes and Biomarker Correlations

Few studies have documented longitudinal cognitive changes in pre‐ataxic SCA individuals. Nigri et al. conducted a 1‐year follow‐up of pre‐ataxic SCA2 individuals and found no significant decline in cognitive scores (eg, MMSE: *P* = 1.0, Digit Span Forward: *P* = 0.30), suggesting stable cognitive function over the short term. In symptomatic SCA2 patients, however, a significant decline was observed in the ROCF (Rey‐Osterrieth Complex Figure)‐copy score (*P* = 0.004).^13^ Conversely, Velázquez‐Pérez et al. reported significant correlations between cognitive test performance and the estimated time to SCA2 ataxia onset: INECO Executive Function score (*P* = 0.027, r = 0.45), IED errors from the CANTAB battery (*P* = 0.016, r = −0.51), and DMS correct responses (*P* = 0.044, r = 0.46), indicating progressive cognitive decline as disease onset approaches. No significant correlation was observed between cognitive performance and CAG repeat size (*P* = 0.771).^16^

Bolzan et al. observed that pre‐ataxic SCA3 patients' cognitive scores were intermediate between HCs and symptomatic individuals, suggesting that cognitive deterioration may begin before the onset of motor symptoms. They found that CCAS‐S total scores correlated with disease progression (TimeToAfterOnset) (*P* < 0.01, rho = −0.512), and affective symptoms also showed a significant correlation (*P* < 0.01, rho = −0.497). No significant associations were found between cognitive scores and CAG repeat size (*P* = 0.313), education level (*P* = 0.497), or depressive symptoms.^17^

Several studies have explored the correlations between cognitive deficits and various biomarkers. Wu et al. reported significant white matter microstructural alterations in pre‐ataxic SCA3 carriers compared to controls (eg, FA reduction in the left inferior cerebellar peduncle, *P* < 0.0001; MD increase, *P* < 0.05) but did not report *P*‐values specifically for correlations between MoCA scores and imaging measures.^19^ Ye et al. demonstrated that loss of cerebellar volume in lobule VI and Crus I correlated with executive and visuospatial deficits in pre‐ataxic and symptomatic SCA3 individuals. Specifically, volume loss in the left lobule VI correlated with poorer COWAT scores (*P* < 0.01, r = 0.52) and JLO scores (*P* < 0.05, r = 0.44); volume loss in the right lobule VI correlated with COWAT scores (*P* < 0.05, r = 0.39); and volume loss in the right Crus I correlated with both COWAT scores (*P* < 0.05, r = 0.44) and PASAT‐2s scores (*P* < 0.05, r = 0.45).^20^

Collectively, these findings underscore early cognitive impairments in pre‐ataxic phases of SCAs, particularly in SCA2, where executive dysfunction and fluency deficits are consistently reported. However, inconsistencies across SCA1, SCA3, and SCA36, combined with the limited availability of longitudinal data, highlight the need for further research.

## Conclusion

This scoping review aimed to map the existing literature, clarify key concepts, and identify research gaps related to cognitive changes in pre‐ataxic individuals with NRE‐SCAs. A total of 13 studies met the inclusion criteria, encompassing SCA1, SCA2, SCA3, and SCA36 patients. The findings confirm that cognitive impairments can be detected before motor symptom onset, particularly in SCA2, where deficits in executive function, processing speed, and verbal fluency are the most consistently reported.^13^, ^16^, ^18^ However, the extent and nature of cognitive dysfunction vary across SCA subtypes. These findings align with prior studies demonstrating that SCAs are not purely motor disorders and that cognitive dysfunction can precede motor symptoms, particularly in SCA2.^13^, ^16^ The presence of cognitive impairment before ataxia onset suggests that early neuropsychological assessments should be integrated into routine evaluations for at‐risk individuals.

Among the different cognitive domains, executive dysfunction emerged as the most prominent alteration, particularly in cognitive flexibility, inhibitory control, and working memory. These deficits were assessed using the TMT‐B, Stroop Interference Task, and phonemic fluency tasks.^16^, ^17^, ^22^ Processing speed impairments, measured using the SDMT, suggest early cognitive slowing, whereas verbal fluency deficits—particularly phonemic fluency—were observed in multiple studies, reinforcing the notion that phonological retrieval deficits precede semantic impairments.^18^

Due to the consistency of executive dysfunction and processing speed deficits in pre‐ataxic SCAs, targeted cognitive testing may facilitate earlier identification of neurodegeneration and improve disease staging. Although the CCAS‐S has been used to assess cognitive impairment in SCAs, its utility in pre‐ataxic individuals remains limited. The CCAS‐S may be useful in identifying group‐level cognitive differences, but its sensitivity in detecting mild cognitive impairments in asymptomatic individuals is uncertain.^15^, ^17^ Therefore, a multimodal assessment strategy—combining traditional neuropsychological tests aimed at the main cognitive alterations observed in SCAs with longitudinal evaluations and neuroimaging biomarkers—appears to be the most promising approach.

These cognitive deficits observed in pre‐ataxic SCAs are likely driven by a combination of cerebellar dysfunction, disrupted cortico‐cerebellar connectivity, and subcortical involvement. The cerebellum plays a crucial role in executive control, working memory, and language processing, and early microstructural alterations in cerebellar pathways have been detected in pre‐ataxic SCA individuals.^19^, ^20^ Studies have reported volume loss in cerebellar lobules VI and Crus I, regions associated with executive function, suggesting that cognitive decline in SCAs may result from disrupted cerebellar–cortical loops rather than isolated cerebellar atrophy.^20^

Beyond the cerebellum, white matter alterations in subcortical tracts and cerebellar peduncles have been documented, indicating that connectivity disruptions between the cerebellum and the prefrontal cortex may underlie executive dysfunction in pre‐ataxic individuals.^19^ Positron emission tomography (PET) studies have reported cerebellar and brainstem hypometabolism, reinforcing the idea that metabolic changes precede structural atrophy and may serve as early indicators of disease progression.^19^ At the molecular level, polyQ protein aggregation in neurons, particularly in pre‐ataxic SCA3 patients, suggests that early cortical dysfunction may contribute to cognitive decline.^17^ Studies using PET and single‐photon emission computed tomography imaging have reported widespread reductions in glucose metabolism, further supporting the hypothesis that functional impairments emerge before significant neuronal loss occurs.^26^

A reanalysis of raw data provided by Ye et al. revealed that pre‐ataxic SCA3 individuals performed significantly better than HCs on tests of visual memory, specifically the BVMT‐R.^20^ This unexpected finding contrasts with the typical pattern of neurodegeneration and suggests the presence of early compensatory mechanisms. Cognitive compensation has been documented in other neurodegenerative diseases, where increased neural efficiency in certain domains temporarily offsets emerging deficits. One possible explanation is plasticity‐driven functional reorganization, in which alternative neural pathways are recruited to maintain cognitive function despite early neurodegeneration.^27^

Additionally, the reanalysis of Ye et al.'s data showed significant deficits in visuospatial processing (JLO) and processing speed (PASAT) in pre‐ataxic SCA3 individuals, reinforcing the hypothesis that cognitive alterations in pre‐ataxic SCAs may not follow a uniform trajectory across all domains.^20^ This dissociation—where deficits emerge in some cognitive functions whereas others remain intact or even enhanced—further highlights the complexity of early‐stage neurodegeneration and suggests that different SCAs may rely on distinct compensatory strategies.

These findings also emphasize the need for more refined cognitive standard screening assessments in pre‐ataxic SCAs. Tools like the MoCA and MMSE failed to detect early differences, whereas domain‐specific tests like the BVMT‐R and PASAT revealed meaningful distinctions. Particularly, Ye et al. used the MACFIMS, previously validated in SCA3, which may offer a more sensitive battery for detecting early cognitive changes in this population.^20^

### Heterogeneity of Findings and Methodological Limitations

Several methodological issues limit the generalizability of findings. Most studies included small sample sizes, reducing statistical power and increasing the risk of type II errors. Methodological heterogeneity was substantial across studies, including differences in cognitive assessment batteries, criteria for defining the pre‐ataxic stage, and follow‐up periods, further complicating comparisons and synthesis. Longitudinal data remain scarce, with only a few studies examining cognitive changes over time. Moreover, cross‐sectional designs dominate the literature, making it difficult to determine whether cognitive deficits worsen progressively or remain stable until motor symptoms emerge.

Certain SCA subtypes, such as SCA36, are underrepresented, limiting the applicability of cognitive findings across the broader spectrum of NRE‐SCAs.^13^, ^14^ Variability in cognitive testing methods and the lack of standardized neuropsychological batteries hinder direct comparisons between studies. Although fluency and executive function tests were widely used, measures of social cognition produced inconsistent results, with some studies reporting deficits and others finding no significant differences. Limited demographic reporting, including education levels and socioeconomic factors, may also introduce bias.^17^, ^21^

Finally, the exclusive inclusion of English‐language publications may have resulted in a language and publication bias, potentially excluding relevant studies published in other languages.

## Future Directions and Research Gaps

Several gaps in the literature must be addressed to clarify the trajectory of cognitive decline in SCAs. Urgently required are longitudinal studies to determine whether early cognitive impairments can predict disease progression and the onset of motor symptoms. Future research should also integrate cognitive testing with advanced neuroimaging techniques such as magnetic resonance imaging, PET, and DTI (Diffusion Tensor Imaging) to correlate structural and functional brain changes with cognitive impairments. This integration can help elucidate the neural mechanisms underlying cognitive decline in SCAs and potentially lead to earlier and more accurate diagnoses.^19^, ^20^

Additionally, larger, multicenter studies are necessary to investigate genotype–phenotype correlations, particularly concerning CAG repeat length and its influence on cognitive decline. Understanding these relationships may help predict disease severity and tailor individualized treatment plans.^17^

The role of psychiatric symptoms in cognitive impairment is another area that remains underexplored. Depression, anxiety, and other neuropsychiatric symptoms are frequently observed in patients with SCAs, yet their impact on cognitive function and disease progression is not well understood. Determining these relationships could lead to more comprehensive treatment approaches that address both motor and cognitive symptoms.^22^

Finally, there are currently no targeted treatments for cognitive impairment in SCAs. Future research should explore various interventions, including cognitive rehabilitation, pharmacological treatments, and noninvasive brain stimulation techniques. These interventions aim to mitigate cognitive decline and enhance the quality of life for patients with SCAs. Developing effective cognitive therapies could also provide insights into the broader mechanisms of neurodegeneration and cognitive dysfunction.^20^, ^22^

In conclusion, this review suggests that cognitive deficits can manifest before motor symptoms in SCAs, with executive dysfunction, verbal fluency deficits, and processing speed impairments commonly reported. The heterogeneity of these findings highlights the urgent need for larger, longitudinal studies, the adoption of standardized cognitive batteries, and the use of multimodal biomarker approaches to delineate the trajectory of cognitive decline in SCAs.

## Author Roles

All authors have contributed equally to the writing and editing of the manuscript.

(1) Research project: A. Conception, B. Organization, C. Execution.

(2) Manuscript preparation: A. Writing of the first draft, B. Review and critique.

R.B.T.: 1A, 1B, 1C, 2A

A.A.V.: 1C, 2A

H.A.G.T.: 1C, 2B

C.H.F.C.: 1B, 1C, 2B

## Disclosures

**Ethical Compliance Statement**: This study did not involve human participants, animal subjects, or identifiable personal data; therefore, approval from an institutional review board (IRB) or ethics committee was not required. The research adhered to established ethical guidelines for conducting scoping reviews and secondary analyses of published literature. As this study did not involve direct patient participation or the collection of patient data, informed consent was not necessary. We confirm that we have read the journal's position on issues involved in ethical publication and affirm that this work is consistent with those guidelines.

**Funding Sources and Conflicts of Interest**: The authors declare that there are no conflicts of interest relevant to this work. The authors declare that no funding was received for the research covered in this article.

**Financial Disclosures for the Previous 12 Months**: The authors declare that there are no additional disclosures to report.

## Supporting information


**Table S1.** Nucleotide repeat expansion‐associated autosomal dominant cerebellar ataxias.
**Table S2.** Characteristics and neurocognitive findings for included studies.
