# Supplementary material for: Cognitive Changes in Pre‐ataxic Spinocerebellar Ataxias: A Scoping Review
Source: Mov Disord Clin Pract. 2025 Jul 14;12(12):2071–9. doi: 10.1002/mdc3.70215 (PMC12715325; doi:10.1002/mdc3.70215)
Supplement: Supplementary file 1 — Table S1. Nucleotide repeat expansion‐associated autosomal dominant cerebellar ataxias. Table S2. Characteristics and neurocognitive findings for included studies. [file MDC3-12-2071-s001.docx]

**Table S1-** Nucleotide repeat expansion-associated autosomal dominant cerebellar ataxias⧫

| **Phenotype** | **Gene** | **Variant type** | **Locus** |
| --- | --- | --- | --- |
| SCA1 | *ATXN1* | (CAG)_n_ repeat | 6p22.3 |
| SCA2 | *ATXN2* | (CAG)_n_ repeat | 12q24.12 |
| SCA3 | *ATXN3* | (CAG)_n_ repeat | 14q32.12 |
| SCA4 | *ZFHX3* | (CAG)_n_ repeat | 16q22.2-q22.3 |
| SCA6 | *CACNA1A* | (CAG)_n_ repeat | 19p13.13 |
| SCA7 | *ATXN7* | (CAG)_n_ repeat | 3p14.1 |
| SCA8 | *ATXN8 /ATXN80S* | (CAG)_n_ / (CTG)_n_ repeat | 13q21/ 13q21.33 |
| SCA10 | *ATX10* | Non-coding (ATTCT)_n_ repeat | 22q13.31 |
| SCA12 | *PPP2R2B* | Non-coding (CAG)_n_ repeat | 5q32 |
| SCA17 | *TBP* | (CAG)_n_ repeat | 6q27 |
| SCA27B | *FGF14* | Non-coding (GAA)_n_ repeat | 13q33.1 |
| SCA31 | *BEAN1* | Non-coding (TGGAA)_n_ repeat | 16q21 |
| SCA36 | *NOP56* | Non-coding (GGCCTG)_n_ repeat | 20p13 |
| SCA37 | *DAB1* | Non-coding (ATTTC)_n_ repeat | 1p32.2-p32.1 |
| SCA51 | *THAP11* | (CAG)_n_ repeat | 16q22.1 |
| DRPLA | *ATN1* | (CAG)_n_ repeat | 12p13.31 |

⧫NRE-SCAs - Adapted from Rudaks et al. (2024)

Abbreviations: NRE: nucleotide repeat expansion. DRPLA: dentatorubralpallidoluysian atrophy. SCA: spinocerebellar ataxia.

**Table S2 -** Summary of characteristics and neurocognitive findings for included studies

| **First author, year, country** | **Pre-ataxic individuals (corresponding SCA)**  **Tests for cognitive assessment**⧫ | **Main cognitive findings for pre-ataxic individuals** |
| --- | --- | --- |
| Nigri, 2020, Italy ^13^ | 13 pre-ataxic **SCA2 \|** Median age: 39y**\|** SARA-s: 0(0-1)**\|** Time to onset of ataxia: 15.5 y (IQR 10.3-18.4)  MMSE**,** DS forward**,** ROCF**,** PVFT**,** SVFT**,** SDMT | -Pre-ataxic SCA2 vs. controls: Preserved intellectual abilities; lower SDMT scores (p=0.011) indicating processing speed & attention deficits.  -No significant differences in other cognitive tests.  -No cognitive decline over a one-year follow-up. |
| Tezenas du Montcel, 2023, France, Germany, USA ^14^ | 14 pre-ataxic **SCA1**\| Median age:39 y\| SARA-s:1(0-2)\|  36 pre-ataxic **SCA3**\| Median age:36y\| SARA-s:1(0-2)  CCAS-S | -Pre-ataxic SCA1 & SCA3 vs. controls: No significant differences (p=0.8; p=1).  -Pre-ataxic SCA1 & SCA3 vs. symptomatic SCA1 & SCA3: Better performance (p=0.005; p=0.0059).  -Definite CCAS: Absent in pre-SCA1, present in 9% of pre-SCA3 patients. |
| Selvadurai, 2024, USA ^15^ | 11 pre-ataxic **SCA1\|** SARA-s: 1.25(sd1.3)\|  15 pre-ataxic **SCA3\|** SARA-s 0.96(sd1.3)\|  Median age:39y**\|** Median education:16y (14-19)  CCAS-S | -Pre-ataxic vs. controls: No significant differences in total or domain-specific CCAS-S scores.  -Pre-ataxic vs. symptomatic SCAs: Better performance on global and domain-specific CCAS-S scores (p < 0.001). |
| Velázquez-Pérez, 2014, Cuba ^16^ | 37 pre-ataxic **SCA2**\| Mean age: 40y(±11)\| SARA-s: 1.07 (sd 1.42)( 0–4)  INECOfs, SCWT, PVFT, EVMT, CANTAB - subsets IED, DMS | -Pre-ataxic SCA2 vs. controls:  Executive dysfunction: lower INECOfs scores (p<0.001), slower SCWT interference times (p=0.021) \|  Verbal fluency deficits: lower PVF scores (p=0.045) \|  Visual memory impairments: fewer DMS correct responses (p=0.017) \| Cognitive flexibility impairments: more IED errors (p=0.009).  - Time to onset of ataxias correlated with INECOfs score (r=0.45; p=0.027)\|  Number of IED errors with preceding EDS (r=−0.51; p=0.016) \|  DMS correct responses correlated (r=0.46; p=0.044) |
| Bolzan., 2024, Brazil^17^ | 23 pre-ataxic **SCA3** with predicted time to onset of ataxia >4y \|Median age: 28 y (sd 5)\| SARA-s: 0.5 (IQR 0–1)  12 pre-ataxic **SCA3** with predicted time to onset of ataxia <4y) \|Median age: 38.9 y (+6.74)\| SARA-s: 1 (IQR 0.5–1.1)  CCAS-S, SCWT, RMET, TMT | -Pre-ataxic SCA3 vs. controls and symptomatic SCA3: Subtle cognitive impairments before motor onset, with intermediate overall performance.  -Most affected domains: Executive function, language, and social cognition.  - Predicted time to onset of ataxia > 4y showed greater impairments, but differences were not statistically significant (small sample size). |
| Rodríguez-Labrada, 2019, Cuba ^18^ | 20 pre-ataxic **SCA2**\| Median age: 36.5y\| Time to onset of ataxia:18y (sd13.6) \| SARA-s: 0.28 (0-1.5)  MMSE, VMT, SCWT, PVFT | -Pre-ataxic SCA2 vs. controls:  Executive dysfunction: Longer SCWT interference times (p < 0.0001)  - No significant differences in PVF, MMSE.  -VMT: More trials required, but statistical significance not confirmed. |
| Wu, 2017 ^19^  China | 23 pre-ataxic **SCA3**\| Median age: 43y (±5.89)\| SARA-s:0.25 (± 0.45)  MoCA | -Pre-ataxia SCA3 vs. controls: No significant differences in cognitive performance.  - Symptomatic SCA3 had lower MoCA scores than pre-ataxic SCA3 and controls (p < 0.01). |
| Ye, 2023, China ^20^ | 30 pre-ataxic **SCA3** \| SARA-s: below 3  MMSE, MoCA  MACFIMS | -Pre-ataxic SCA3 vs. controls: Better visual memory (BVMT-R p<0.0001–0.002)\| Worse visuospatial performance (JLO p<0.0001)\| Processing speed deficits (lower PASAT-3s p=0.004 & PASAT-2s p=0.017)\| Altered memory retrieval (more CR insertions, p=0.001).***  -No significant differences in CVLT-II, COWAT, MMSE, or MoCA.*** |
| Elyoseph ,2023, Israel^21^ | 5 pre-ataxic **SCA3** \|Median age: 35 y (±4)  RMET, TAS-20, FBt | - Pre-ataxic SCA3 vs. controls: Similar results on RME, TAS-20, and FBt (normal mentalizing). |
| Martínez-Regu, 2020, Spain ^22^ | 11 pre-ataxic **SCA36** \|Median age: 42 y \|Median education: 13y (±4) \|SARA-s: 0.73 ± 1.1  MMSE, FAB, TMT-B, SCWT, WCST, WAIS-III (DS), JLO, BNT-15, PVF, SVF | -Pre-ataxic SCA36 vc. controls: Impaired phonological fluency (PVF p=0.037)\| Deficits in executive function & processing speed (TMT-B p=0.007).  - Global cognition: Normal; MMSE & FAB show no clinically relevant deficits. |
| Hengel, 2023, Germany^23^ | 43 pre-ataxic **SCA3** \|SARA-s: 1 (range:0–2)  MoCA | -Pre-ataxic SCA3 vs. controls: No significant difference (p=0.14).  -Mild cognitive impairment in 15% of controls, 25% of pre-ataxic SCA3 and 31% of symptomatic SCA3.  -Moderate/severe MoCA impairment: Absent in pre-ataxic SCA3 and controls. |
| Klinke, 2010, Germany^24^ | 2 pre-ataxic **SCA1** \|SARA-s: 0  SCT,SRT,CRT,PVFT, RI, ICRT, Hanoi, VLMT, FLT,SFT, MWT-B,BNT-15 | -Pre-ataxic SCA1: typical results across all cognitive, emotional, and motor tests.  -Symptomatic SCA1 had broader cognitive impairments, with deficits identified in 33% of cognitive test parameters, mainly frontal attentional and executive functions. |
| Li., 2024, China^25^ | **2**4 pre-ataxic **SCA3**  MMSE | -Pre-ataxic SCA3: 2/24 were cognitively impaired (MMSE-s <24).***  - Symptomatic SCA3: 44/295 were cognitively impaired. |

Notes: ***Indicates unpublished data; the raw data was sent by the corresponding authors upon request.

Abbreviations: Ed: Education. Mdn: Median. IQR: Interquartile Range. N: Number. HC: Healthy Controls. PAO: predicted age of ataxia onset. SARA-s: Scale for the Assessment and Rating of Ataxia score. Y: years.

⧫ Abbreviations (Tests Used): BNT-15: Boston Naming Test - 15-item version; BVMT-R: Brief Visuospatial Memory Test - Revised; CANTAB: Cambridge Neuropsychological Test Automated Battery; CCAS (-S): Cerebellar Cognitive Affective Syndrome (-Scale); COWAT: Controlled Oral Word Association Test; CRT: Choice Reaction Time Task; CVLT-II: California Verbal Learning Test - Second Edition; DMS: Delayed Matching to Sample Test (CANTAB subset); DS: Digit Span; EDS: extradimensional set; EVMT: Evoked Verbal Memory Test; FAB: Frontal Assessment Battery; FBt: False Belief Test; FLT: Figural Learning Tasks; Hanoi: Tower of Hanoi Test; ICRT: Inverse Choice Reaction Task; IED: Intradimensional/Extradimensional Set Shift Test (CANTAB subset); INECOfs: INECO Frontal Screening Test; JLO: Judgment of Line Orientation Test; MACFIMS: Modified Minimal Assessment of Cognitive Function in Multiple Sclerosis; MMSE: Mini-Mental State Examination; MoCA: Montreal Cognitive Assessment; MWT-B: Mehrfachwahl Wortschatz Interferenztest (German Multiple Choice Vocabulary Test); PASAT (-2s or 3s): Paced Auditory Serial Addition Tes (- 2 or 3 seconds)t; PVFT: Phonemic Verbal Fluency Test; RI: Response Inhibition; RMET: Reading the Mind in the Eyes Test; ROCF: Copy and delayed recall of the Rey-Osterrieth Complex Figure; SCT: Symbol Counting Test; SCWT: Stroop Color-Word Test; SDMT: Symbol Digit Modalities Test; SVFT: Semantic Fluency Test; SRT: Simple Reaction Time Task; TAS-20: Toronto Alexithymia Scale 20-item version; TMT-A/B: Trail- Making Test A & B; Token Test: Linguistic performance test assessing comprehension; VLMT: Verbal Learning Memory Test (Rey Auditory Verbal Learning Test); WAIS-III (DS): Wechsler Adult Intelligence Scale, Third Edition (WAIS-III) Digit Span subtest. WCST: Wisconsin Card Sorting Test.
